# MRI of atypical lipomatous tumor: does contrast help? A multicenter study

**DOI:** 10.1007/s00256-025-04957-8

**Published:** 2025-06-05

**Authors:** Hande Nalbant, Yasser G. Abdelhafez, Cyrus Bateni, Felipe Godinez, Sonia Lee, Michelle Zhang, Jinyi Qi, Nimu Yuan, Fatma Sen, Ahmed W. Moawad, Khalid M. Elsayes, Morgan A Darrow, Thomas M. Link, Michele Guindani, Lorenzo Nardo

**Affiliations:** 1 Department of Radiology, University of California Davis, Sacramento, CA USA; 2 Radiology and Nuclear Medicine Department, South Egypt Cancer Institute, Assiut University, Assiut, Egypt; 3 Department of Radiological Sciences, University of California Irvine, Irvine, CA USA; 4 Department of Diagnostic Radiology, McGill University Health Center, Montreal, Canada; 5 Department of Biomedical Engineering, University of California, Davis, CA USA; 6 Department of Diagnostic Imaging, University of Texas MD Anderson Cancer Center, Houston, TX USA; 7 Department of Diagnostic Radiology, Mercy Catholic Hospital, Darby, PA USA; 8 Department of Pathology, University of California Davis, Sacramento, CA USA; 9 Department of Radiology and Biomedical Imaging, University of California San Francisco, San Francisco, CA USA; 10 Department of Biostatistics, University of California Los Angeles, Los Angeles, CA USA

**Keywords:** Lipoma, Atypical lipomatous tumor, MRI, Gadolinium

## Abstract

**Objective:**

To compare the diagnostic performance of non-contrast MRI versus MRI with contrast for differentiating atypical lipomatous tumors (ALT) from lipomas.

**Materials and methods:**

This multicenter retrospective study included subjects with a histopathologic diagnosis of lipoma or ALT and a preoperative MRI study with contrast. An experienced musculoskeletal radiologist reviewed the images in two sessions, the first session with non-contrast only, and the second session, including postcontrast sequences. In each session, the radiologist assigned a binary classification (lipoma or ALT) and a 5-point diagnostic score to reflect clinical reporting. Pathology reports served as the reference standard. McNemar’s test was used to evaluate the statistical significance of the differences in sensitivity and specificity, while intraclass correlation coefficients (ICC) compared ordinal diagnostic scores.

**Results:**

Four-hundred and forty-one patients (219 ALT, 222 lipoma) were eligible for analysis. In the first session, 76.1% of the lesions were classified as true negative and 76.3% as true positive. In the second session, 74.8% of lesions were assessed as true negative and 77.2% as true positive. There were no significant differences between the two readings in terms of sensitivity or specificity. The agreement in assigning the exact score between the two sessions, as measured by ICC, was 0.878 (95%CI: 0.855–0.898).

**Conclusion:**

Our study found no significant difference between the radiological readings of MRIs that used only precontrast sequences were used for the evaluation of lipoma and ALT, and those that included both pre- and postcontrast-enhanced sequences.

## Introduction

Lipomatous soft tissue tumors are the most common mesenchymal neoplasms [1]. Most of which are lipomas and do not need further interventional procedures, unless symptomatic or for cosmetic reasons [2]. Even if they are deep seated with larger sizes, they have no potential for malignancy [1, 3]. On the other hand, well differentiated liposarcomas (WDLs) or atypical lipomatous tumors (ALTs) represent about 50% of all liposarcomas and might need wide surgical excision with negative surgical margins due to the potential of recurrence and rarely dedifferentiation and metastatic potential [4–6]. The World Health Organization (WHO) classifies ALT and WDL into the same category, but they differ in their anatomical location and resectability. ALTs originate in the extremities or trunk and, generally, if possible they are resected with curative intent and wide surgical margin. On the other hand, WDLs arise in the mediastinum or retroperitoneum; sites where complete surgical excision cannot always be achieved. This could increase the chances for local recurrence, dedifferentiation, and even distant metastasis [7, 8].

At the present time only pathologic analysis of the whole lesion ensures the correct lesion characterization. Typically, lipomas have mature adipocytes with no cellular atypia or pleomorphism. Unlike lipomas, ALTs consist of mature adipocytes with atypical hyperchromatic nuclei. ALTs often contain fibrous septa in which these atypical cells might be difficult to identify. Moreover, these atypical cells may be focal and could be missed in non-representative biopsies. A molecular technique using fluorescence in situ hybridization (FISH) that examines the application of murine double minutes (MDM2) is the current most sensitive test for diagnosing ALT. Since they have different behavior and management, a non-invasive differentiation of ALTs from lipomas is crucial [9, 10].

MRI is the standard imaging modality for the evaluation of lipomatous lesions [1]. In many practices, it is common to systematically acquire additional MR sequences after injection of gadolinium-based contrast agent (GBCA) to differentiate between benign and malignant lipomatous soft tissue tumors [11]. The administration of GBCAs increases acquisition time, cost, and procedure complexity (e.g., requiring radiologist coverage). In addition, administration of GBCAs is associated with immediate adverse reactions with intensity ranging from mild (e.g., hives) to severe and rarely fatal side effects, including anaphylactic shock. Other known GBCAs side effects include nephrogenic systemic sclerosis [12] and accumulation in different tissues, e.g., bone and brain [13, 14]. Several authors argue that GBCA may not help distinguish between lipoma and ALT [11, 15–19], although these studies are not conclusive due to small sample sizes or study design limitations. Therefore, the question related to the utility of GBCA administration in all lipomatous lesions remains to be answered.

The aim of this work is to compare the diagnostic performance of MRI without contrast to MRI with and without GBCA for differentiating ALT from lipoma in a large multicenter dataset, which uses pathologic assessment as the gold standard.

## Materials and methods

### Study population

This was a retrospective multicenter study involving five institutions in North America. The study protocol was approved by the local Institutional Review Board (#1,213,041) and the collection and distribution of images was approved and regulated by the University Reliance System and Data Transfer Agreements. Consent was waived for this retrospective chart review.

Patients were included if they had a pathologically proven surgically resected lipoma or ALT and underwent a preoperative MRI study with contrast within 3 months from surgical resection. The exclusion criteria were incomplete imaging studies, low-quality images (such as motion artifact, poor fat suppression, metallic artifacts), studies acquired on scanners with magnetic field strength lower than 1.5 Tesla, prior surgery, and a nonconclusive pathology report for lipoma or ALT.

### MRI protocol

MRI studies were performed on more than 20 different scanners including: GE (Discovery MR750, MSK extreme, Optima MR450w, Signa excite, Signa genesis, Signa HDe, Signa HDx, Signa HDxt), Siemens (Aera, Avanto, Espree, Skyra, Sonata, Symphony Tim, Verio and Trio Tim), Philips (Achieva dStream, Gyroscan intera, Ingenia), and Toshiba Titan. Scanners operated at either 1.5 or 3.0-Tesla magnetic field strength. The MRI imaging protocol included nonfat-suppressed T1-weighted fast spin echo (FSE) and T2-weighted fat-suppression (FS) FSE or STIR sequences with sagittal, coronal, and axial T1 SE and T2 FS FSE as well as pre- and post-contrast T1-weighted FS images. At least one pre- and one post-contrast series were required for the current evaluation. DICOM images were anonymized at each participating institution and exported for central reading at our institution.

### Radiologic evaluation

All images were independently reviewed by a senior musculoskeletal radiologist with more than 10 years of experience in musculoskeletal tumor imaging. The radiologist was blinded to any clinical data. The images were reviewed in two stages. At each stage, the radiologist reported the individual qualitative features of the lesion under question (e.g., architectural complexity, presence/absence of septa thicker than 2 mm, level of fat suppression), then the overall impression was recorded as a binary decision (L or ALT), and the radiologist’s confidence level about that binary impression was captured on a 4-point scale ranging from one (least confident) to four (most confident). Furthermore, to mimic the clinical reporting language, the probability of being ALT or lipoma was reported on a 5-point diagnostic scale as previously published where 1 = consistent with L, 2 = probably L, 3 = equivocal, 4 = probably ALT, and 5 = consistent with ALT [11].

In the first stage, only sequences without contrast ([WOC], non-contrast T1-weighted and fluid sensitive conventional sequences) were evaluated. After a gap of at least 1 month, the second stage was started employing reading of all the available sequences, including sequences with contrast (WOC&WC). The median interval between the first and second readings for a given scan was 161 days (range: 31–199 days).

### Reproducibility analysis

During the second round, ~ 14% of all the studies, all with no post-contrast sequences, that were not included in the final diagnostic accuracy analyses, were re-evaluated by the same radiologist to establish intra-observer reproducibility.

For measuring inter-observer reproducibility, a subgroup of the current cohort (*n* = 185, 42%) was interpreted by another radiologist (3-year experience in MSK imaging), following the same exact reporting protocol. To evaluate inter-reader agreement, we referred to a prior study’s data; 185 of the 441 patients have been previously reported in that prior study [14] where the diagnostic performance of MRI for discriminating lipoma from ALT was established. In the current work with a bigger cohort, we specifically focused on GBCM utility and compared the diagnostic performance of MRI WOC to MRI WOC&WC for differentiating ALT from lipoma. The readings from one of the radiologists in a subset of that data (*n* = 185, 42%), which follows the same exact reading protocol (Images were reviewed in two stages. At each stage the radiologist reported the individual qualitative features of the lesion, then the overall impression as a binary decision was recorded, and the radiologist’s confidence level about that binary impression was captured on a 4-point scale, finally the probability of being ALT or lipoma was reported on a 5-point diagnostic scale) were utilized in the current submitted work to evaluate inter-observer agreement.

### Pathologic analysis

Pathology reports served as the standard of reference for each case. Lesions consisting of mature adipose tissue with uniform adipocytes resembling normal fat were diagnosed as SLs. On the other hand, lesions with hyperchromatic cytologically atypical cells which often had a multinucleate floret-like appearance were diagnosed as ALTs. Those cells typically were located within fibrous bands or near vessels or both. Additionally, more variation in the size, shape, and appearance of adipocytes as well as the presence of lipoblasts were considered suggestive of ALTs.

When the histologic findings were equivocal, a second pathologist was consulted, or FISH testing was performed to evaluate for the presence of MDM2 gene amplification. Equivocal histologic findings were identified as the presence of fibrous bands or adipocyte variation without identifiable atypical cells. Moreover, if a tumor was in a deep location or had imaging findings suggestive of ALT but the microscopic appearance resembled lipoma, it also was sent for MDM2. The MDM2 gene is usually amplified in ALT but not in lipoma and this was detected using FISH.

### Statistical analysis

Cohen’s kappa test was used for assessing the degree of agreement between the binary reading decisions for the same radiologist (intra-observer reproducibility) and between the two radiologists (inter-observer reproducibility).

True-positive (TP), true-negative (TN), false-positive (FP), and false-negative (FN) readings were identified based on histopathologic validation. Accordingly, diagnostic performance indices were calculated in the form of sensitivity, specificity, accuracy, positive predictive value (PPV), negative predictive value (NPV), and likelihood ratios (LRs). The nonparametric McNemar’s test was used to evaluate the statistical significance of the differences in sensitivity and specificity between the paired readings (WC&WOC). Intraclass correlation coefficient (ICC) was used to compare the ordinal diagnostic scores assigned for each reading.

The area under the curve (AUC) from receiver operating characteristic (ROC) analysis was used to demonstrate overall accuracy of the 5-point diagnostic score. Continuous data were summarized as mean and standard deviation or median (range). Categorical variables were summarized as frequency and percentages. Confidence intervals (95% CI) were reported where applicable. In all analyses, *P*-value < 0.05 (two-tailed) was considered statistically significant. The analysis was generated using R (R Core Team,Vienna, Austria), the Real Statistics (Real Statistics Resource Pack software [Release 9.0]) and MedCalc (MedCalc Software, Mariakerke, Belgium).

## Results

### Study population

**Fig. 1:**
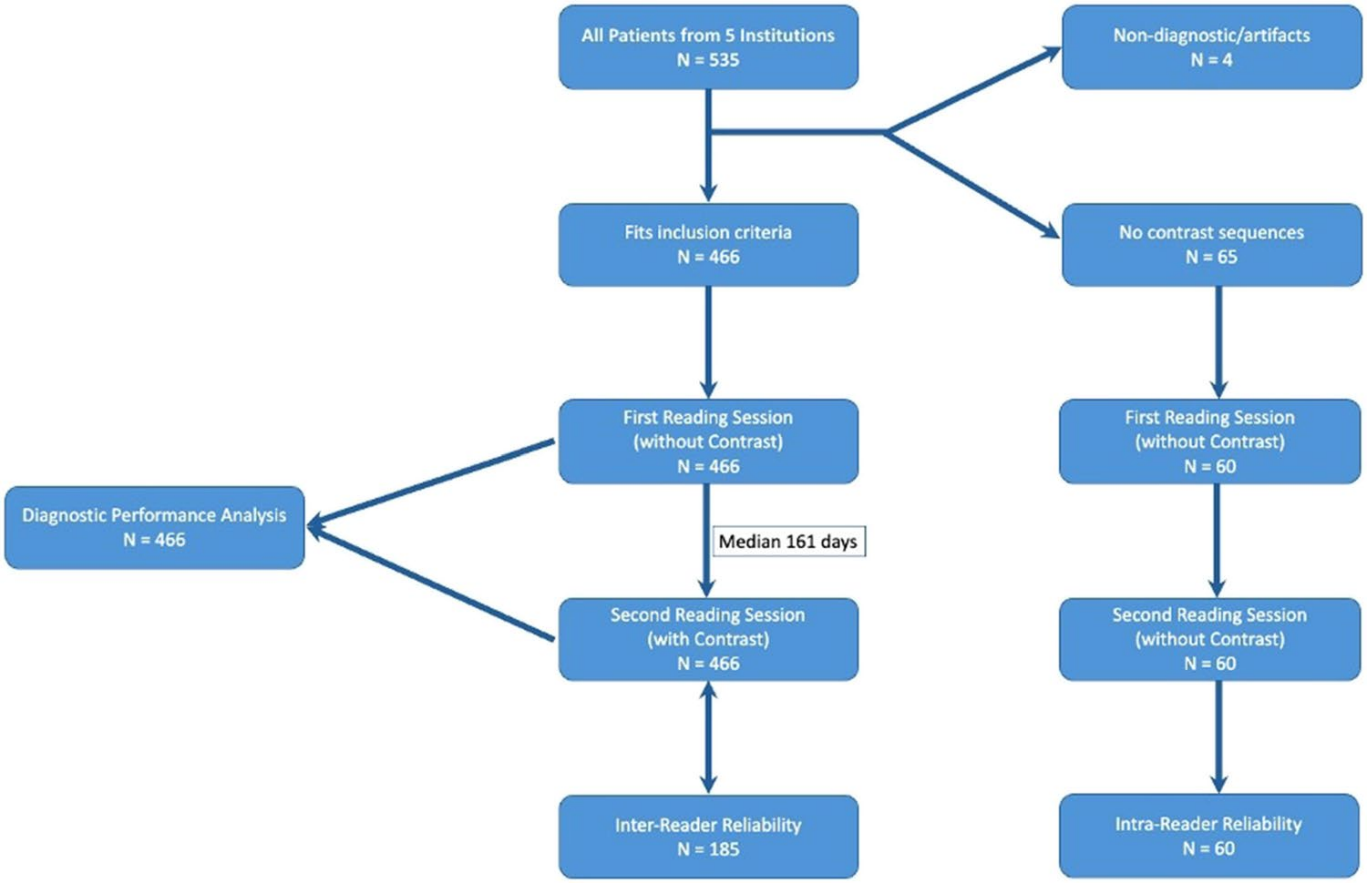
Flowchart of the study population

Five-hundred-thirty-five patients had a preoperative MRI and a pathologically proven lipoma or ALT; of these 94 patients were excluded (Fig. 1). Specifically, 65 patients had no postcontrast images, 25 patients were scanned with scanners with field strengths lower than 1.5 Tesla, 2 patients had nondiagnostic or incomplete imaging and 2 patients with non-identified lesions. The enrolled cohort eligible for analysis consisted of 441 patients (235 female, 206 males; median age 58 [23–89] years). Of them, 219 (49.7%) lesions were histopathologically proven ALTs and 222 were SLs. Detailed patient characteristics are shown in Table 1.

**Table 1:** Summary of patients’ characteristics

Characteristics		*n*	Percentage (%)
Age	Mean ± SD	58.2 ± 12.0	
	Median range	58	(23–89)†
Sex	Female	235	53.3
	Male	206	46.7
Presentation	Incidental	37	8.4
	Not incidental	402	91.6
Pain or discomfort	No	227	51.9
	Yes	210	48.1
Surgery	Excisional biopsy	53	12.0
	Resection	388	88.0
Free margin	Negative	142	46.0
	Positive	167	54.0
MDM2 status	Negative	77	45.6
	Positive	92	54.4
Final pathology	ALT	219	49.7
	Lipoma	222	50.3

All data are presented as frequency and percentage except age. Data for the remaining patients (if < 441) was not available

†If age is > 89, it was recoded as 89 years per IRB requirements

### Reproducibility analysis

The intra-observer reproducibility, measured from 60 precontrast studies that were not included in the subsequent analysis, was substantial (0.69: 95%CI: 0.51–0.88). The inter-observer agreement for classifying 185 (42%) scans as lipoma vs. ALT was also substantial for WC and WOC (respectively, 0.63; 95%CI: 0.52–0.75 and 0.69; 95%CI: 0.58–0.79) for WOC. The readings of the whole cohort (*n* = 441) by a single radiologist were used for the rest of the current analysis.

### Radiologic impression with and without contrast

**Fig. 2:**
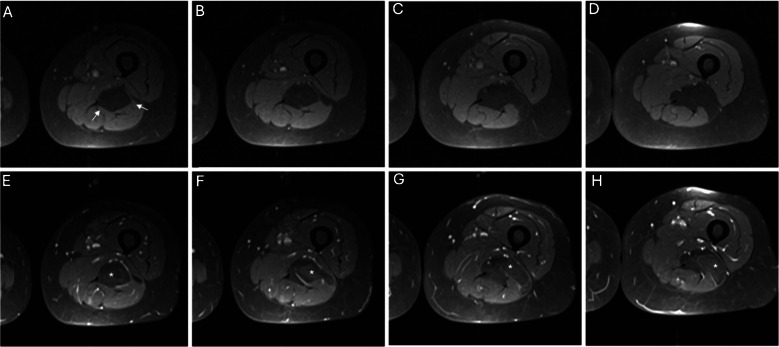
MRI of a left thigh deep seated lipomatous lesion (arrows). Precontrast axial T1 WI-FS (**A**, **B**, **C**, **D**) demonstrate complete fat suppression of the lesion with similar architecture complexity compared to the adjacent subcutaneous fat. The radiologist’s binary impression was lipoma in the first reading session. Postcontrast axial T1 WI-FS (**E**, **F**, **G**, **H**) demonstrate heterogenous internal contrast enhancement (asterisk). The impression was changed as ALT in the second reading session. (The radiologist’s 5-point diagnostic scores were equivocal for both sessions). The histopathology results revealed that the lesion is ALT

In the WOC session, 221 lesions were assessed as lipomas, of those 76.5% were true negative and 23.5% were ALTs (false negative). An example of false negative is given in Fig. 2. Conversely, 220 lesions were assessed as ALTs; of those, 75.9% were true positive, and 24.1% were lipomas (false positive).

In the WOC&WC session, 216 lesions were assessed as lipomas, of those 76.9% were true negative and 23.1% were false negative. Also, 225 lesions were assessed as ALTs; of those 75.1% were true positive and 24.9% were false positive. Detailed diagnostic performance indices are shown in Table 2.

**Table 2:** Diagnostic performance indices of the two MR reading sessions in 441 patients, and for superficial, and deep lesions

	All lesions		Superficial lesions		Deep lesions	
Measure	WC	WOC&WC	WC	WOC&WC	WC	WOC&WC
True positive	167	169	8	12	159	157
True negative	169	166	99	98	70	68
False positive	53	56	10	11	43	45
False negative	52	50	15	11	37	39
Sensitivity% (95%CI)	76.3 (70.1–81.7)	77.2 (71.0–82.6)	34.8 (16.4–57.3)	52.2 (30.6–73.2)	78.7 (72.4–84.2)	77.7 (71.4–83.3)
Specificity% (95%CI)	76.1 (70.0–81.6)	74.8 (68.5–80.4)	90.8 (83.8–95.5)	89.9 (82.7–94.9)	65.4 (55.6–74.4)	63.55 (53.7–72.6)
Positive predictive value% (95%CI)	75.9 (71.1–80.1)	75.1 (70.4–79.3)	44.4 (26.2–64.4)	52.2 (35.5–68.4)	81.1 (76.6–84.9)	80.1 (75.6–83.9)
Negative predictive value% (95%CI)	76.5 (71.7–80.7)	76.9 (73.0–81.1)	86.8 (83.0–90.0)	89.91 (85.3–93.2)	61.95 (54.7–68.7)	60.2 (53.0–70.0)
Positive likelihood ratio (95%CI)	3.19 (2.50–4.09)	3.06 (2.41–3.88)	3.79 (1.68–8.55)	5.17 (2.61–10.24)	2.28 (1.74–2.98)	2.13 (1.64–2.77)
Negative likelihood ratio (95%CI)	0.31 (0.42–0.40)	0.31 (0.24–0.39)	0.72 (0.53–0.97)	0.53 (0.35–0.82)	0.33 (0.24–0.44)	0.35 (0.26–0.47)

The numbers of true positive, true negative, false positive, and false negative are given as frequencies

Among the histopathologically diagnosed ALT, 11 out of 167 (6.6%) true positive readings on WOC were interpreted as lipoma on WOC&WC readings. On the other hand, among histopathologically diagnosed L, 14 out of 169 (8.3%) true negative WOC readings were falsely interpreted as ALT after contrast; however, there was no significant difference between the two readings regarding sensitivity or specificity (Table 3).

**Table 3:** Agreements of radiologist’s binary impressions on the two reading sessions for all lesions, superficial, and deep lesions

	Radiologist’s impression	With and without contrast		Kappa (95% CI)
	Without contrast	Lipoma	ALT	
All	**L** (Actual pathology: L, ALT)†	194 (155, 39)	27 (14, 13)	0.78* (0.72–0.84) (Substantial)
	**ALT** (Actual pathology: L, ALT)†	22 (11, 11)	198 (42, 156)	
Superficial	**L** (Actual pathology: L, ALT)†	105 (94, 11)	9 (5,**4**)	0.63* (0.44–0.81) (Substantial)
	**ALT** (Actual pathology: L, ALT)†	4 (4, **0**)	14 (6,8)	
Deep	**L** (Actual pathology: L, ALT)†	89 (61, 28)	18 (9,9)	0.74* (0.66–0.82) (Substantial)
	**ALT** (Actual pathology: L, ALT)†	18 (7, 11)	184 (36,148)	

McNemar’s test was performed between pairs of radiologist’s impressions (without contrast vs. with and without contrast) and the overall difference was not statistically significant (*P* > 0.05) when all lesions were considered, and so for the superficial and deep lesions

†Data in parentheses reflect the interpretation breakdown according to the histopathology reference standard

*The level of agreement was also measured for the histopatholically-diagnosed L and ALT and was respectively 0.70 (CI: 0.59–0.81) and 0.69 (CI: 0.58–0.81) for all lesions; 0.53 (CI: 0.25–0.80) and 0.66 (CI: 0.37–0.94) for superficial lesions; and 0.70 (CI: 0.57–0.84) and 0.67 (CI: 0.54–0.81) for deep lesions

On further analysis by lesion depth, the performance of the two readings was comparable for superficial and deep lesions (Table 2); however, the WC readings correctly categorized 4 superficial lesions as ALT. Though this change might be clinically meaningful, no statistically significant difference was noted for sensitivity or specificity (Table 3).

Furthermore, readings WOC&WC did not result in any significant change of the confidence levels compared to WOC only readings (respectively, 3.3 ± 0.9 vs. 3.3 ± 0.8; *P* = 0.83).

The overall agreement on assigning confidence scores about this binary reading was 0.54 (95%CI: 0.48–0.61) with no significant difference between the average confidence scores between the two sessions.

### Five-point diagnostic scores

**Fig. 3:**
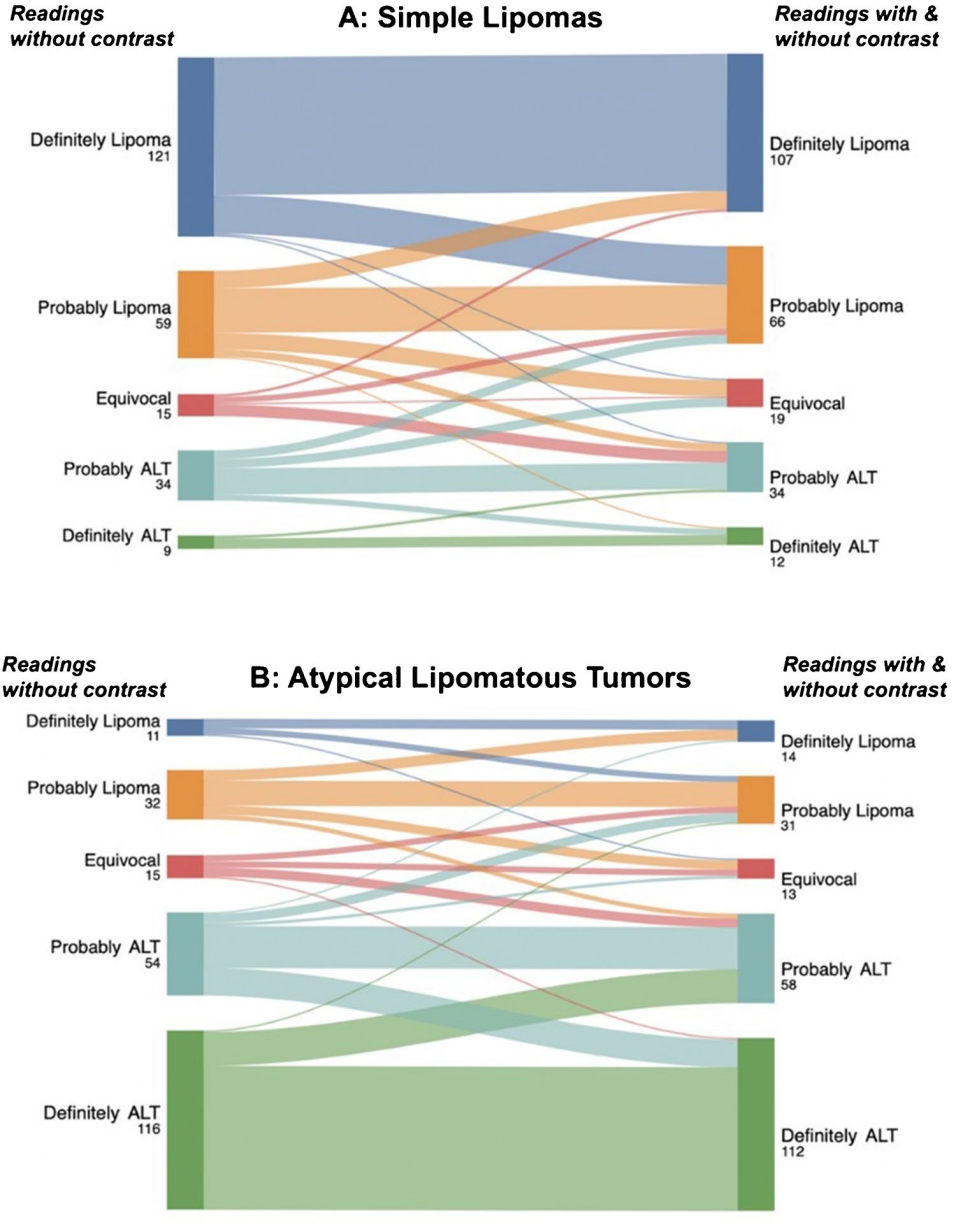
Sankey diagram demonstrating changes in the 5-point diagnostic scores between two reading sessions for the pathologically proven lipomas (**A**) and atypical lipomatous tumors (**B**)

There was good agreement on assigning the exact score between the two sessions, as measured by ICC (0.88; 95%CI: 0.86–0.90) [20]. The overall accuracy of the diagnostic scores was slightly higher for WOC (AUC = 0.86 [95%CI: 0.82–0.89]) compared to WOC&WC (AUC = 0.83 [95%CI: 0.79–0.87]) (*P* = 0.051). A detailed illustration of WOC diagnostic scores reassignment on readings is shown in the diagram in Fig. 3.

**Fig. 4:**
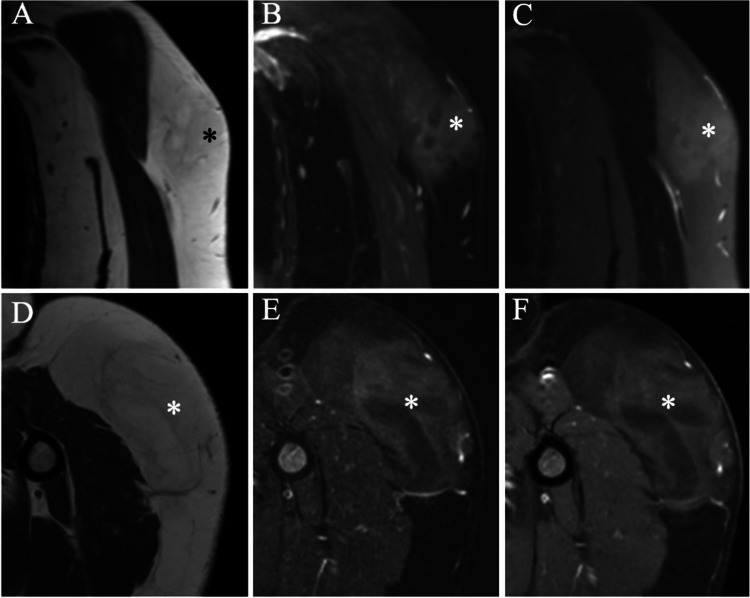
MR images of a huge superficial left shoulder lipomatous lesion (asterisk). Coronal (**A**) and axial (**D**) T1 WI demonstrate a fatty lesion with heterogeneous intensity and ill-defined borders. Coronal (**B**) and axial (**E**) T2 W-FS and coronal STIR sequence (**C**) indicate that the lesion has partial fat saturation. Axial postcontrast T1 WI-FS shows diffuse contrast enhancement. The lesion was interpreted as equivocal in both reading sessions. The histopathology confirmed a diagnosis of L with negative MDM2

**Fig. 5:**
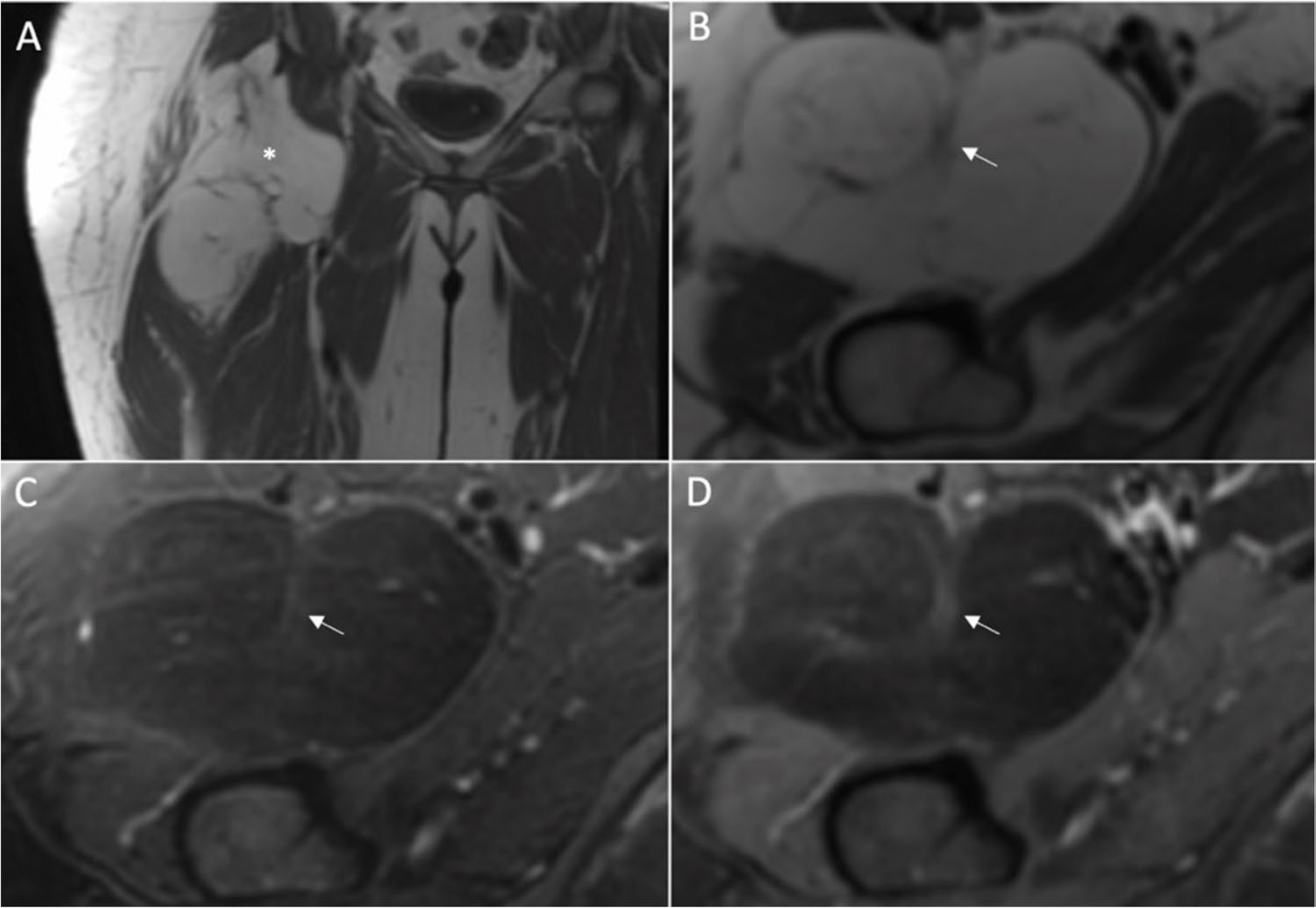
MRI of a big, deep-seated right hip lipomatous lesion (asterisk). Coronal (**A**) and axial(**B**) T1 WI demonstrate the lesion has thick septae (arrow) and T2 WI-FS (**C**) demonstrate complete fat saturation of lesion. Post-contrast T1 WI-FS demonstrate septal enhancement. The lesion was reviewed as equivocal in first reading session and changed false positively as ALT in the second reading. The lesion was histopathologically diagnosed as lipoma

The frequency of equivocal interpretations (Score 3) did not vary between the two sessions (29 for WOC, vs. 32 for WOC&WC; *P* > 0.05). An example of equivocal category is given in Fig. 4. Twenty-nine cases were interpreted as equivocal on the WOC reading session, after the inclusion of the postcontrast images the radiologist changed scores of 24 of them to either lipoma (*n* = 9, 4 of them were false negative) or ALT (*n* = 15, 8 of them were false positive). Examples of changes in the equivocal category are given in Fig. 5. Table 4 summarizes the changes from and to the equivocal categories in both pathologies.

**Table 4:** Changes of radiologist’s interpretation from equivocal category to other diagnostic scores

Pathology	Without contrast	With and without contrast		McNemar’s *P*-value
		Not equivocal (Scores 1,2,4,5)†	Equivocal (Score 3)	
All pathologies	Not equivocal (Scores 1,2,4,5)†	385	27 (2,17,8,0)	0.78
	Equivocal (Score 3)	24 (1,8,14,1)	5	
Lipoma	Not equivocal (Scores 1,2,4,5)†	190	18 (1,11,6,0)	0.47
	Equivocal (Score 3)	13 (1,4,8,0)	1	
ALT	Not equivocal (Scores 1,2,4,5)†	195	9 (1,6,2,0)	0.82
	Equivocal (Score 3)	11 (0,4,6,1)	4	

†Data in parentheses represent the breakdown of the conversion from or to the equivocal category to other diagnostic scores

### Changes in key qualitative features

**Fig. 6:**
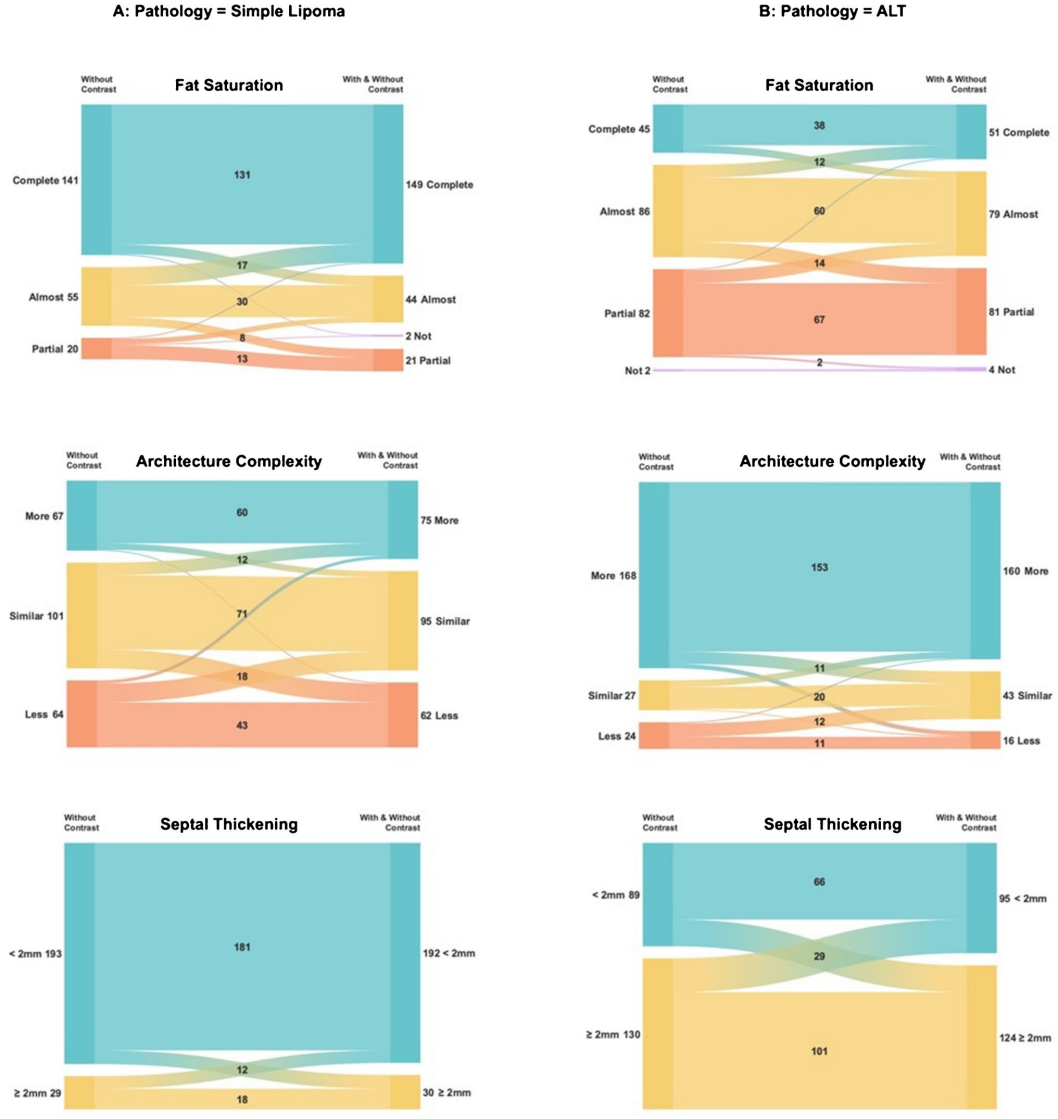
Sankey diagrams demonstrating changes in interpreting fat saturation, architecture complexity, and septal thickening of pathologically proven SLs (**A**) and ALTs (**B**) between the two reading sessions

**Fig. 7:**
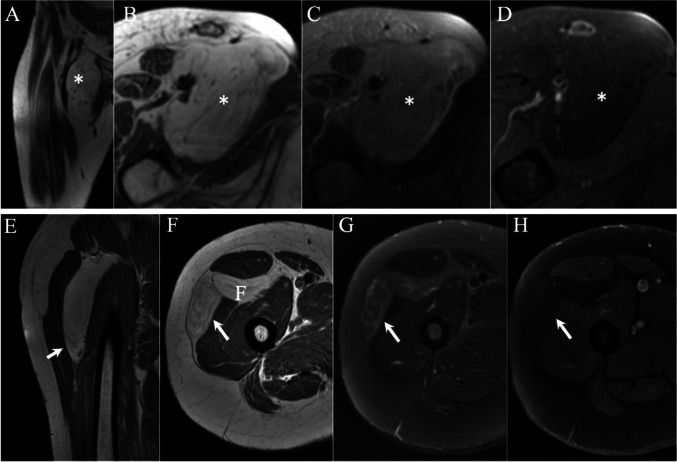
MRIs of two different deep seated, proximal lower limb lipomatous lesions (asterisk and arrow). Coronal (**A**) and axial (**B**) T1 WI demonstrate similar/more complex architecture with thin septae, T2 WI-FS (**C**) shows complete fat saturation, and axial postcontrast T1 WI-FS demonstrates no contrast enhancement in the first case. Coronal (**E**) and axial (**F**) T1 WI show a lesion with more complex architecture in its lowermost part (arrow), compared to the adjacent subcutaneous fat. T2 WI-FS (**G**) demonstrates hyperintense septae, and postcontrast axial T1 WI-FS (**H**) demonstrates no contrast enhancement. Both lesions were proved to be SLs on histopathology with negative MDM2. The two examples were interpreted as ALT (false positive) in the first reading without contrast and correctly identified as lipomas on the second reading with contrast

A summary of key qualitative feature recategorization between the two reading sessions is shown in Fig. 6. Architectural complexity and septal thickness were the two most frequently changed features after contrast injection. Among pathologically proven lipomas, 15 (9.1%) out of 165 that were previously reported as similar/less architectural complexity on first reading session were changed to more complex in the second reading; of those 3 were finally miscategorized as ALTs. On the other hand, 7 cases out of 57 (12%) pathologically proven lipomas and previously assessed as having more complex architecture were recategorized on the second reading as having less or similar level of architecture complexity as the surrounding fat; 2 of those were correctly classified as lipomas (Fig. 7).

**Fig. 8:**
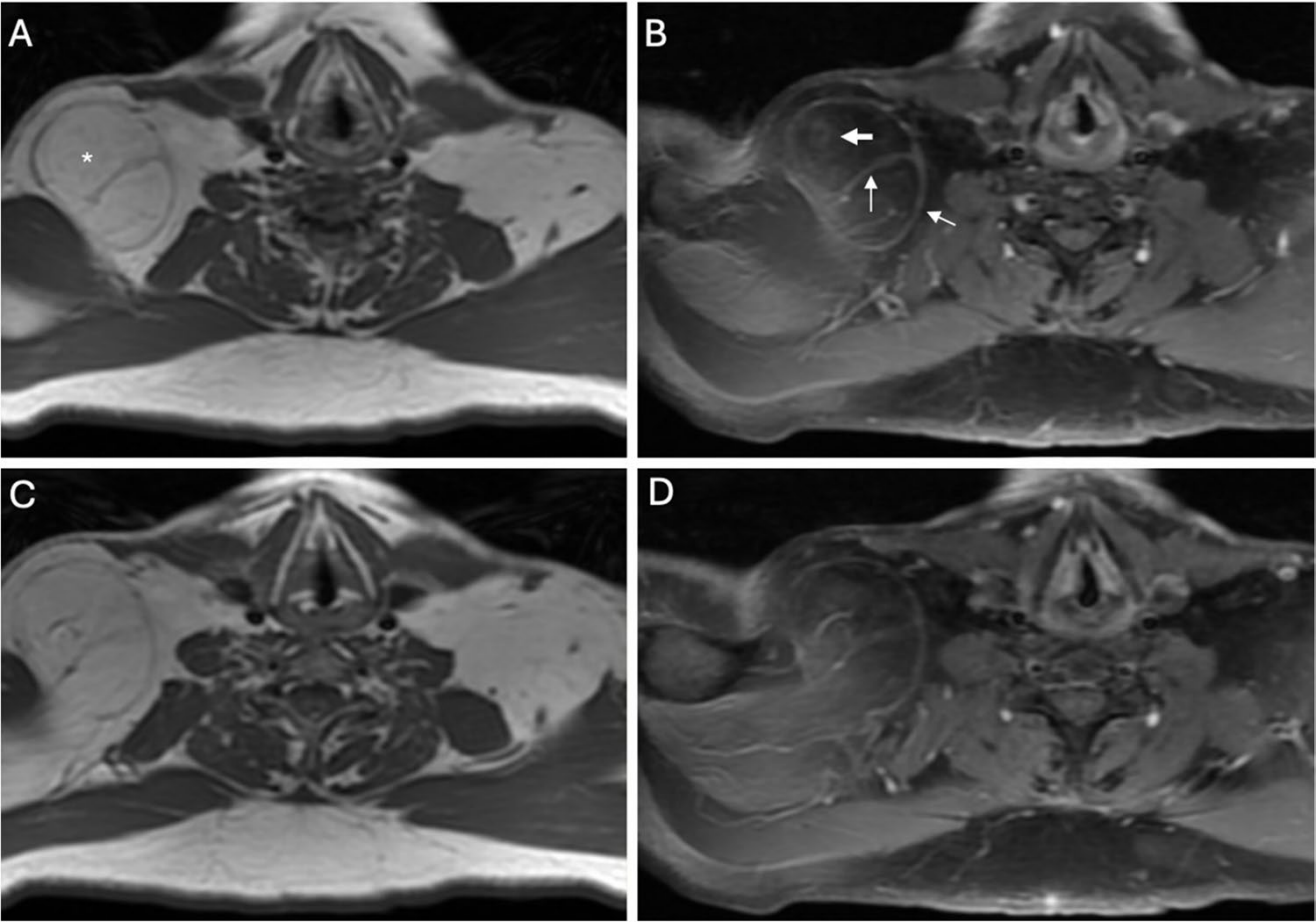
MRI of a right shoulder superficial seated lipomatous lesion (asterisk). Axial T1 WIs (**A**, **B**) demonstrate similar architecture with few septa compared to adjacent subcutaneous fat. The lesion was scored as probably lipoma in the first reading session. Postcontrast axial T1 WI-FS (**B**, **D**) demonstrates internal contrast enhancement of the septa and capsule (arrows). The score was changed as probably ALT in the second session. The histopathology results revealed with positive MDM2 that the lesion is ALT

Among pathologically proven ALTs 15 out of 168 (8.9%) cases were changed from more complex architecture to similar or less complex in the second session; of them 6 were ultimately miscategorized as lipomas. Seven out of 51 (13.7%) cases previously assessed as having less or similar complexity were recategorized as more complex architecture in the second session and 3 of them were interpreted correctly as ALTs**.** Among ALTs, septa were recategorized in 29 patients changed from thick on first session to thin septa on second session; 4 were finally miscategorized as lipomas. Among lipomas, 12 patients changed from thin septa on first session to thick; of them, 4 were miscategorized as ALTs. Figure 8 demonstrates an example of a pathologically proven ALT, septa were changed from thin to thick on second session and it was finally interpreted correctly as ALT.

## Discussion

In this multicenter study, we found that the inclusion of gadolinium-based postcontrast MRI sequences for the radiological readings was comparable to readings with non-contrast MRI sequences alone in the differential diagnosis of lipoma and ALT.

Atypical lipomatous tumors need to be excised with negative surgical margins due to local recurrence risk, rare potential of dedifferentiation and rare possibility of generating metastases [21, 22]. The surgical procedure may be associated with complications [4, 10]. On the other hand, benign lipomas do not need excision, unless symptomatic; however, these lesions are often surgically removed because of the difficulties in differentiating them from their malignant counterpart pre-surgically [10, 11, 16, 17, 23, 24].

Contrast enhancement is a marker of increased vascular permeability and angiogenesis, which often correlate with malignant tumorous microenvironment [25]. For this reason, it is recommended in the assessment of soft tissue masses including lipomatous lesions. However, for the differentiation between ALT and lipoma there is still no consensus on using contrast.

Several studies focused on septal enhancement and reported that contrast enhancement might be helpful for differentiation of ALTs from lipomas [16–19]. Cheng et al. developed a scoring system, with one of the parameters being “the presence of nodular enhancement more than 1 cm or fat saturation less than 75%.” This parameter was found to be a significant predictor for differentiation of the two pathologies [24]. Nagano et al. also developed another scoring system and found that contrast enhancement is an indicator for ALTs [26]. The main limitation of those studies is the small simple sizes. On the other hand, Panzarella and Shannon worked with a slightly larger group and reported that gadolinium enhancement does not have an additional effect on this differential diagnosis [23, 27]. Moreover, Nardo et al. reviewed 246 (70 of them were ALTs) patients and reported that postcontrast images may cause misdiagnosis in a few cases [11]. To the best of our knowledge our study represents the largest sample size with 441 patients (219 ALT). When we compared the binary impressions of two independent readings WOC and WOC&WC, performed by the same MSK radiologist, we found no significant difference in sensitivity or specificity. Moreover, when we examined the 5-point diagnostic scale, which closely resembles the language used in clinical practice, we found that reviewing cases without postcontrast images had slightly higher, though not significant, overall accuracy compared to reviewing postcontrast images. These findings could signify that conventional MRI without contrast might be as accurate and adequate for diagnosis of ALT/lipoma differentiation compared to MRI with contrast.

Typical MRI appearance of a grossly fatty mass, with no or few thin septa, no or minimal areas of enhancement and full fat signal suppression on fat saturation images is thought to be specific for lipomas [15]. On the other side of the spectrum, a deep-seated lesion, large in size, with thick irregular/nodular septa, and prominent foci of high signal on fat saturation fluid sensitive sequences suggests an ALT [11, 24]. However, in daily practice, the radiologist encounters many lesions with equivocal MRI appearance. In our study the radiologist reported 29 (6.6%) of all cases as equivocal in the first session (WOC). After including postcontrast images, 41.4% of those with an initial equivocal reading was changed to a true diagnosis, 41.4% changed to a false diagnosis, and 17.2% remained equivocal. Additionally, the radiologist changed 27 (6.6%) of previously diagnosed cases (Scores 1–2 or Scores 4–5) to equivocal (Score 3). Postcontrast images did not help the radiologist to decrease the number of equivocal reports or give more accurate decisions for previously equivocal ones.

The most serious late adverse reaction of GBCA is nephrogenic systemic fibrosis, which is currently known to have no specific or consistently effective treatment [28]. Lately, it has also been demonstrated that gadolinium may persist in some organs including the brain even years after GBCAs administration [29]. The consequences of this accumulation are still unknown. Accordingly, one of the radiologist’s main goals is to reduce unnecessary administration of these contrast agents. In this work, we tried to demonstrate how contrast media could affect radiologist’s decisions for evaluating lipomatous tumors, specifically lipomas and ALTs.

Our study has some limitations. First, the current study primarily reported the readings from a single radiologist. Though we demonstrated substantial intra- and inter-reader reproducibility for 14% and 42% of the current cohort, respectively, future work could employ readers from different institutions with varying levels of training and experience before considering the generalizability of the current findings. Second, the radiologist was exposed to the same set of images before the second round of reading; this exposure, though blinded to the pathology, might affect their second decisions not only because of the inclusion of postcontrast images, but also because of potential recall. However, the two reading sessions had a minimum of 30 days and a median of 186 days which comfortably mitigated the possibility of this effect. Another limitation is that the binary nature of the pathologies in this study does not reflect the real clinical practice. In clinical practice, lipomatous lesions encompass a large number of heterogeneous tumors beyond the simple dichotomization as lipoma or ALT. However, these two types are the most commonly encountered in daily practice. Additionally, while the reader was blinded to the breakdown of lipoma and ALTs, the sample had a nearly 50/50 breakdown of these two pathologies, which is not reflective of the relative frequencies in clinical practice. This potentially could have influenced the interpretation results.

As the sole reader was a musculoskeletal radiologist who interprets exams regularly for the evaluation of lipomas and ALTs and receives pathologic feedback on many exams, the overall experience may not accurately reflect those of all radiologists. Lastly, the dataset was available only in anonymized format; therefore, it was not possible to retrieve any additional data regarding patients. This could have prevented answering several ancillary questions, such as specific types of GBCA.

In conclusion, our study demonstrated no significant differences between MRI radiologic readings with and without contrast in the assessment of lipoma and ALTs. While more studies may further elucidate the role of gadolinium in this specific context, our results should be considered when protocolling MRI scans, especially in light of possible gadolinium known and unknown side effects.

## Data Availability

The datasets analyzed during the current study are not publicly available because the subjects did not provide written consent for their data to be publicly shared.
